# Computational models for the assessment of manufactured nanomaterials: Development of model reporting standards and mapping of the model landscape

**DOI:** 10.1016/j.comtox.2018.12.002

**Published:** 2019-02

**Authors:** L. Lamon, D. Asturiol, A. Vilchez, R. Ruperez-Illescas, J. Cabellos, A. Richarz, A. Worth

**Affiliations:** a European Commission, Joint Research Centre (JRC), Ispra, Italy; b Leitat Technological Center, c/de la Innovació 2, Terrassa, Barcelona, Spain

**Keywords:** Model reporting template, Reporting standard, QSAR, QSPR, PBK, Environmental exposure model

## Abstract

Different types of computational models have been developed for predicting the biokinetics, environmental fate, exposure levels and toxicological effects of chemicals and manufactured nanomaterials (MNs). However, these models are not described in a consistent manner in the scientific literature, which is one of the barriers to their broader use and acceptance, especially for regulatory purposes. Quantitative structure-activity relationships (QSARs) are *in silico* models based on the assumption that the activity of a substance is related to its chemical structure. These models can be used to provide information on (eco)toxicological effects in hazard assessment. In an environmental risk assessment, environmental exposure models can be used to estimate the predicted environmental concentration (PEC). In addition, physiologically based kinetic (PBK) models can be used in various ways to support a human health risk assessment. In this paper, we first propose model reporting templates for systematically and transparently describing models that could potentially be used to support regulatory risk assessments of MNs, for example under the REACH regulation. The model reporting templates include (a) the adaptation of the QSAR Model Reporting Format (QMRF) to report models for MNs, and (b) the development of a model reporting template for PBK and environmental exposure models applicable to MNs. Second, we show the usefulness of these templates to report different models, resulting in an overview of the landscape of available computational models for MNs.

## Highlights

• The transparent and systematic reporting of computational models facilitates their regulatory acceptance and use.
• A reporting format for physiologically based kinetic, toxicodynamic and environmental fate models was developed.
• The QSAR Model Reporting Format (QMRF) was adapted to describe QSARs for nanomaterials.
• The model documentation is stored in the publicly accessible JRC Data Catalogue.
• The model documentation was used to give an overview of the model landscape for nanomaterials.

## 1 Introduction

Different types of mathematical models have been developed for quantification of exposure values or effect concentrations in the process of chemical risk assessment,^1^ and are potentially useful to support legislation, such as the EU regulation on the Registration, Evaluation, Authorisation and Restriction of Chemicals (REACH) [1].

REACH addresses chemical substances and manufactured nanomaterials (MNs). MNs are defined by the European Commission (EC) *Recommendation on the Definition of a nanomaterial* as materials containing particles, in an unbound state or as an aggregate or as an agglomerate and where, for 50% or more of the particles in the number size distribution, one or more external dimensions is in the size range 1–100 nm [2].

Quantitative structure-activity relationships (QSARs) are predictive models based on the assumption that the activity of a substance is related to its structure. The concept is well established and in the past decade it has been applied more efficiently and extensively due to the availability of chemicals databases and to the encouragement to use computational methods for providing required information for REACH. Quantitative structure-property relationships (QSPRs) are conceptually the same as QSARs but they relate structure to physicochemical properties of chemicals.

According to REACH Annex XI, QSARs may be applied as alternative methods to animal testing in filling data gaps. The condition to be met for accepting QSARs in regulatory decision making are, among others, that the scientific validity of the model(s) has been established and an adequate documentation of the method used is provided. According to the OECD Guidance Document on the Validation of (Quantitative) Structure-Activity Relationship (QSAR) models [3], a QSAR is considered reliable and applicable if it fulfils the five principles for the validation of QSARs: 1) addresses a defined endpoint, 2) consist of an unambiguous algorithm, 3) has a defined domain of applicability, 4) appropriate measures of goodness-of-fit, robustness and predictivity, and 5) a mechanistic interpretation [4]. For regulatory applications, QSARs should be documented following the QSAR Model Reporting Format (QMRF), an internationally harmonised template for summarising and reporting key information on QSAR models, including the results of any validation studies [5]. The QMRF is used to submit QSAR models to the QSAR Model Database, which is a publicly accessible database maintained by the Joint Research Centre intended to help identify valid QSARs that can be used to support the regulatory assessment of chemicals (https://qsardb.jrc.ec.europa.eu/qmrf/). A first step to adapt the QMRF to report QSARs developed for MNs was proposed in eNanoMapper [6], [7].

Physiologically based toxicokinetic models (PBK) are numerical models commonly derived from physiologically relevant compartments and processes and constructed from mass-balance equations (i.e. accounting for material entering and leaving a system). In the context of REACH, they are considered relevant in human health risk assessment according to the Chapter R8 of the *Guidance on information requirements and chemical safety assessment on the characterisation of dose[concentration]-response for human health* [8], where it is recognised that PBK models can support the derivation of derived non-effect level (DNEL) from animal data to account for human health risk. PBK models can be used to determine or adjust specific assessment factors (AFs): 1) route-to-route, 2) interspecies and 3) high-dose-low-dose extrapolation. In addition, PBK modelling data can aid in the quantification of intraspecies variability, denoted by variation in anatomical, physiological and biochemical parameters with age, gender, genetic predisposition and health status. With a view to replacing animal testing, PBK models can be used to extrapolate from effect levels observed in *in vitro* systems to the *in vivo* situation, often referred to as (quantitative) *in vitro* to *in vivo* extrapolation.

Environmental exposure modelling is an important aspect in risk assessment and it includes material flow and environmental fate models, which are used for the calculation of a predicted environmental concentration (PEC) [9]. This definition of environmental exposure modelling will be used throughout the manuscript. These models treat the environment as a complex system composed of different compartments (i.e. air, water, soil, etc.), and compute the mass flows between the different compartments and between regions, taking into consideration the emissions of chemicals.

Although environmental exposure and PBK models are considered in the ECHA guidance as supporting information requirements in chemical safety regulation [8], they are less consistently reported in the literature compared to QSARs and QSPRs. In fact, there is no official template or set of rules to consider the validity of an environmental exposure model. For reporting PBK more consistently, the European Committee for Standardisation (CEN) published a CEN Workshop Agreement on *Standard documentation of chemical exposure models* to provide a framework for model description which would facilitate the use and comparability of models, addressing the minimum information to be provided for documenting PBK models [10], [11]. The proposed standard aims at promoting appropriate model application, thus supporting informed decision making. In addition, work is ongoing within the OECD to develop guidance for the characterisation and reporting of PBK models.

Within the drug development regulatory field, US Food and Drug Administration (FDA) and European Medicines Agency (EMA) have published draft guidelines on how to report PBK models and their simulations [12], [13]. These templates are specific to drug design, and aimed at supporting the pharmaceutical industry in complying with regulatory requirements. Accordingly, these guidelines depend on the regulatory requirements in force, and are highly focused on processes related to traditional chemicals (such as metabolism) and therefore are not fully applicable to the MNs and regulatory applications such as supporting hazard or risk assessment of MNs.

Furthermore, specific to environmental exposure models, Buser et al. [14] reported a wide variability on the application of the same model (EUSES, the recommended model for drafting the chemical safety report in REACH [15]) in regulatory submissions, and suggested six principles for good modelling practice that would allow a more consistent use of multimedia fate models and communication of the results.

The objective of this manuscript is twofold. First, we propose model reporting templates for systematically and transparently reporting MN models that are suitable to support regulatory assessments. The model reporting templates include (a) the adaptation of the QMRF to report models applicable to MNs, and (b) the development of a novel model reporting template for PBK and environmental exposure models applicable to MNs. Second, we report the models available in the literature with the model reporting templates and use them to describe the current landscape of computational models for MNs.

## 2 Methods

### 2.1 Development of the model templates

We have defined two different model reporting templates for our scope. One is defined for QSARs/QSPRs, and consists in the adaptation of the QMRF (https://qsardb.jrc.ec.europa.eu/qmrf/) to report models applied to MNs. The second template, defined for PBK and environmental exposure models, takes inspiration from experience with the QMRF, from lessons learned from the CEN workshop agreement [10], [11] and from good modelling practices identified by Buser et al. [14]. We reported PBK-type and environmental exposure models in the same template because they are both multicompartment models and focus on predicting the fate and transport of a MN in the body or in the external environment. A review on the PBK models populating the inventory is presented in Lamon et al. [16].

### 2.2 Bibliographic searches

We first conducted a bibliographic search on available QSPRs and QSARs applied to MNs. We then documented models in a searchable Excel file (i.e. the reporting model template, hereafter inventory). Finally, we described the “landscape” of available QSPR/QSARs, PBK and environmental exposure models, including the endpoints and MNs, the descriptors that are mostly considered in the models as well as the data sources used, the processes taken into consideration, and model limitations and assumptions.

More details on the bibliographic searches are available in Worth et al. [17].

## 3 Results

### 3.1 Templates for reporting computational models

#### 3.1.1 Modification of the QMRF to report MN models

The QMRF is inspired to the OECD principles on QSAR model validation [3] and was proposed to give visibility to QSARs for regulatory applications. We updated the existing QMRF by adding fields relevant to report MNs (e.g. shape, size, surface coating).

**Fig. 1:**
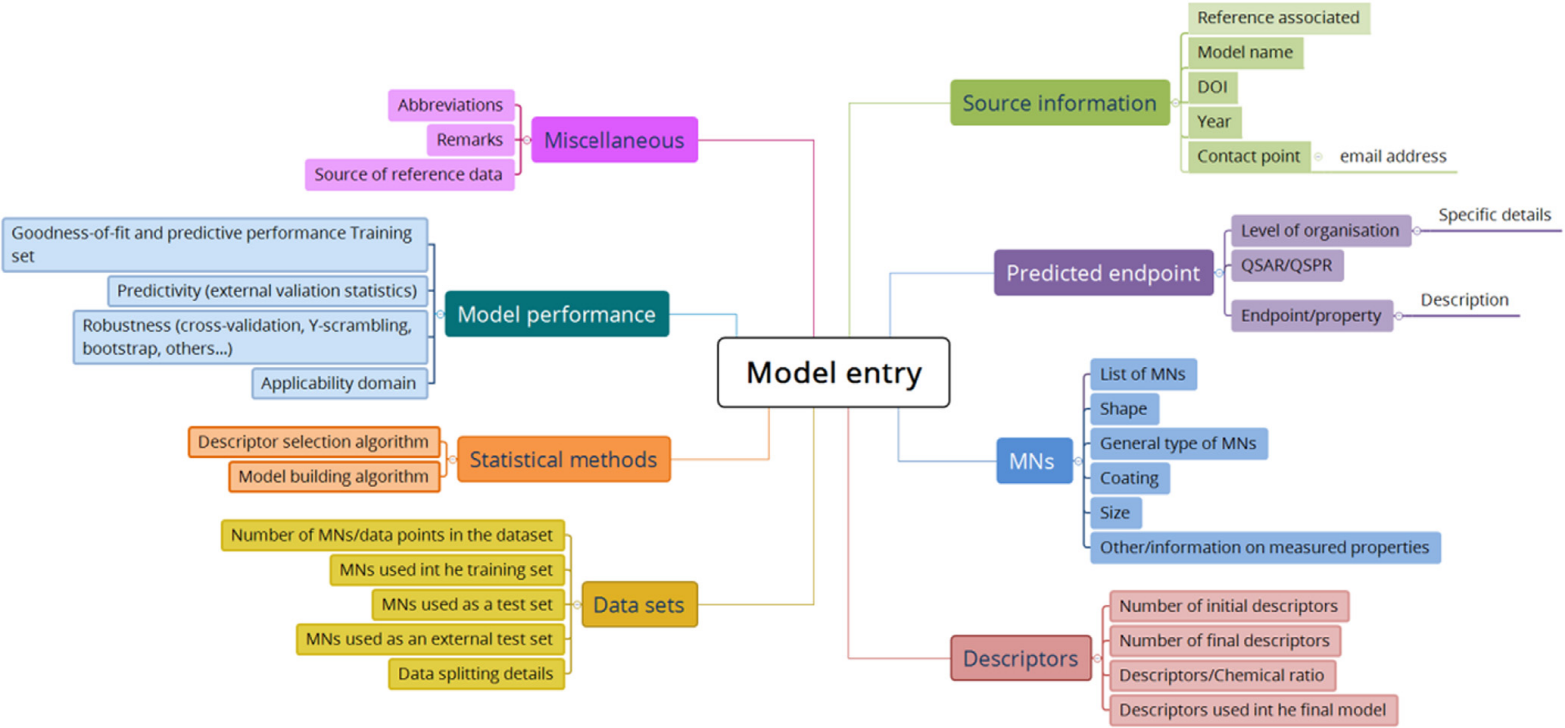
Map of the information provided in the QSAR/QSPR model template.

Fig. 1 shows the structure of the inventory, covering all different sections and corresponding parameters used to categorise the available models. The content of each section is summarised below.

1. **Source Information**: reference details of the publication, contact author and model name. When no model name was available, the predicted endpoint and the method used to generate it were used instead.
2. **Predicted Endpoint**: information about the type of cells or organisms used in the study, the toxicity endpoint, and whether it is predicting an *in vitro* or *in vivo* activity (QSAR), or information about the physicochemical properties (QSPR).
3. **MNs**: in this section, first a more generic description of the type of MNs considered in the model is provided (e.g. metals, metal oxides, carbon-based or polymeric nanomaterials); and second, a more specific definition of the MNs is defined (e.g. material composition, Ag), including physicochemical properties such as size, shape or surface functionalisation.
4. **Descriptors**: this section specifies the number and type of descriptors used in the model (e.g. fingerprints, topological, geometric, physicochemical). It also includes the ratio MNs/descriptors, which is a value used to assess the complexity of the model and possible overfitting [18].
5. **Data sets**: size of the training, test and external validation data sets are given. Details of data splitting used in the model building phase are also specified when applicable.
6. **Statistical methods**: an explanation of the descriptor selection process (screening from an initial set to obtain a reduced final set of descriptors) as well as the details of the statistical method applied to generate the model are provided. The software tools are also specified if they were provided in the publication.
7. **Model performance**: this section contains four inputs corresponding to the principles 3 and 4 of the OECD guideline, i.e. applicability domain, goodness-of-fit, robustness and predictivity.
8. **Miscellaneous**: it includes the definition of abbreviations, references to the datasets used for modelling and relevant remarks, e.g. mechanistic interpretation (principle 5 of the OECD guideline).

#### 3.1.2 Definition of a template for reporting PBK-type models and environmental exposure models

In this paper, we aim at developing a template that is generic enough to include not only descriptions of PBK models but also environmental exposure models that can be applied in chemical risk assessment.

The model reporting template presented by Chiffroy et al. [11], based on the standard reporting protocol for exposure models developed in CEN [10] is more suitable for our purpose. In fact, the model template developed in this manuscript reflects the CEN levels 1 to 5 of knowledge to describe the PBK model, but requires less detail compared to the model reporting template proposed by CEN. One of the intended uses of the model reporting format and the inventory proposed in this manuscript is to use it as a screening tool for model selection or model judgement according to the list of MNs covered by a certain model, the parameters involved, the model type (e.g. steady state, dynamic), the level of validation (based on experimental studies or not) and the reported uncertainties and assumptions.

**Fig. 2:**
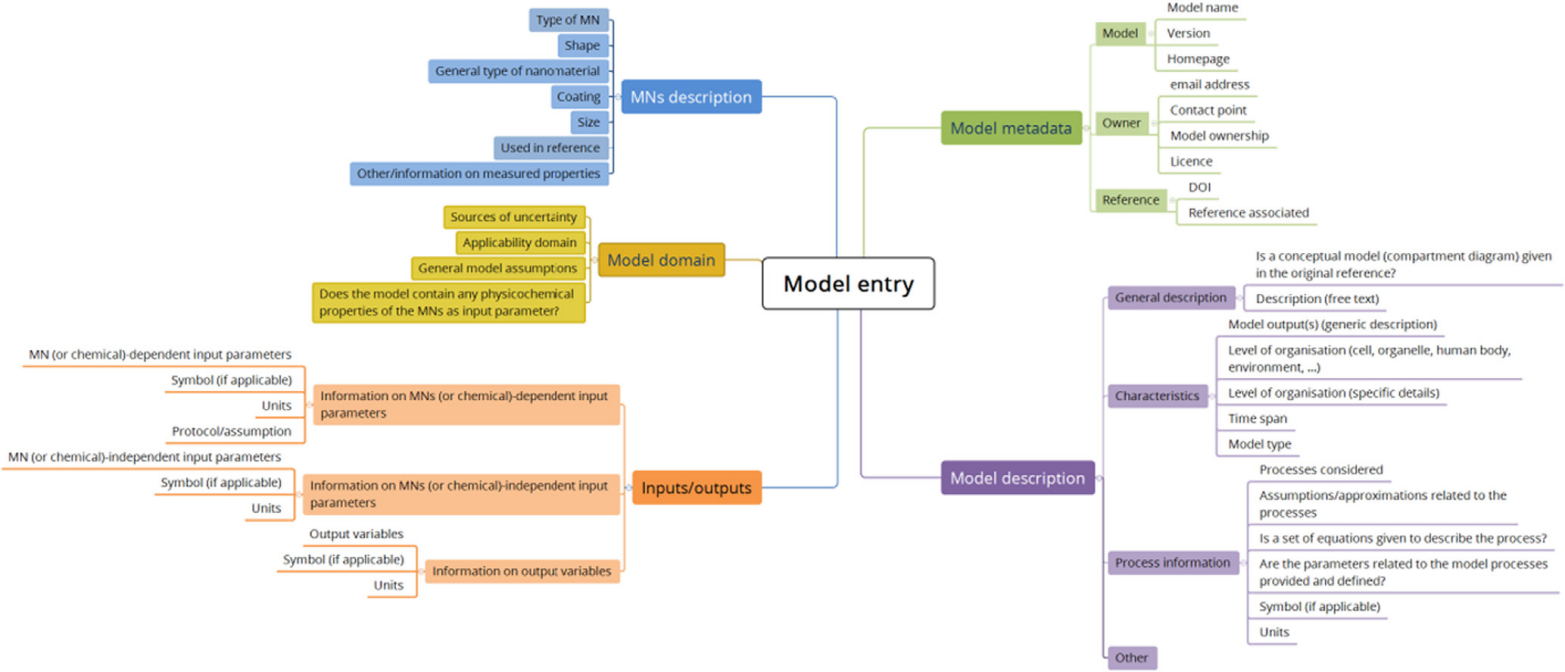
Inventory labels included in the inventory for characterising PBK and environmental exposure models.

To account for environmental exposure models, the template was double-checked against the six principles of good modelling practice identified by Buser et al. [14]. The resulting model reporting template is summarised in Fig. 2. The content of each section is summarised below.

1. **Model metadata:** gives information on the model version, software version and contact information.
2. **Model description:** gives information on the type of model, a generic description of the outputs, the level of organisation (cellular level, organ level, organism, or environment), the model type and the temporal resolution of the model; processes included in the model are listed as well as assumptions and approximations made. If available, the parameters used to describe the processes are also listed.
3. **Input/output parameters:** input parameters are listed as NM-dependent or independent. Output parameters are also specified.
4. **MN description:** when a model is applied to MNs, these are described following the list of nanospecific parameters considered in the QSAR model reporting template (e.g. type of MN, size, shape, coating); information on measured properties is flagged when such information was used.
5. **Model domain:** this label contains information on the applicability domain, general model assumptions, whether the model includes nanospecific input parameters as model inputs, and identifies sources of uncertainty.

### 3.2 Model inventories

After a careful review of the search results, a total of 59 publications for QSAR models (out of around 800 found in the initial search) and 29 publications for QSPR models (out of around 350 found in the initial search) were included in the final inventory.

Our search on PBK and environmental exposure models applied to MNs led to the compilation of 474 papers. 48 publications on PBK models were selected for the inventory, as were 81 publications on environmental exposure models. The number of entries in the inventory is larger than the number of publications because sometimes one paper reports more than one model or application.

The resulting inventories of QSAR/QSPR and PBK/environmental exposure models are freely available as .xls files through the JRC Science Hub (https://ec.europa.eu/jrc/en/science-update/review-computational-models-safety-assessment-nanomaterials) and as part of the ECVAM collection in the JRC Data Catalogue (http://data.jrc.ec.europa.eu/dataset/jrc-eurl-ecvam-nanocomput).^2^

### 3.3 Landscape of computational models applied to MNs

The compiled inventories were analysed to draw conclusions on the availability of computational models on QSARs/QSPRs and environmental exposure models applied to MNs. The content of the inventory on PBK models is reported in the review by Lamon et al. [16].

#### 3.3.1 Availability of QSAR and QSPR models applied to MNs

**Fig. 3:**
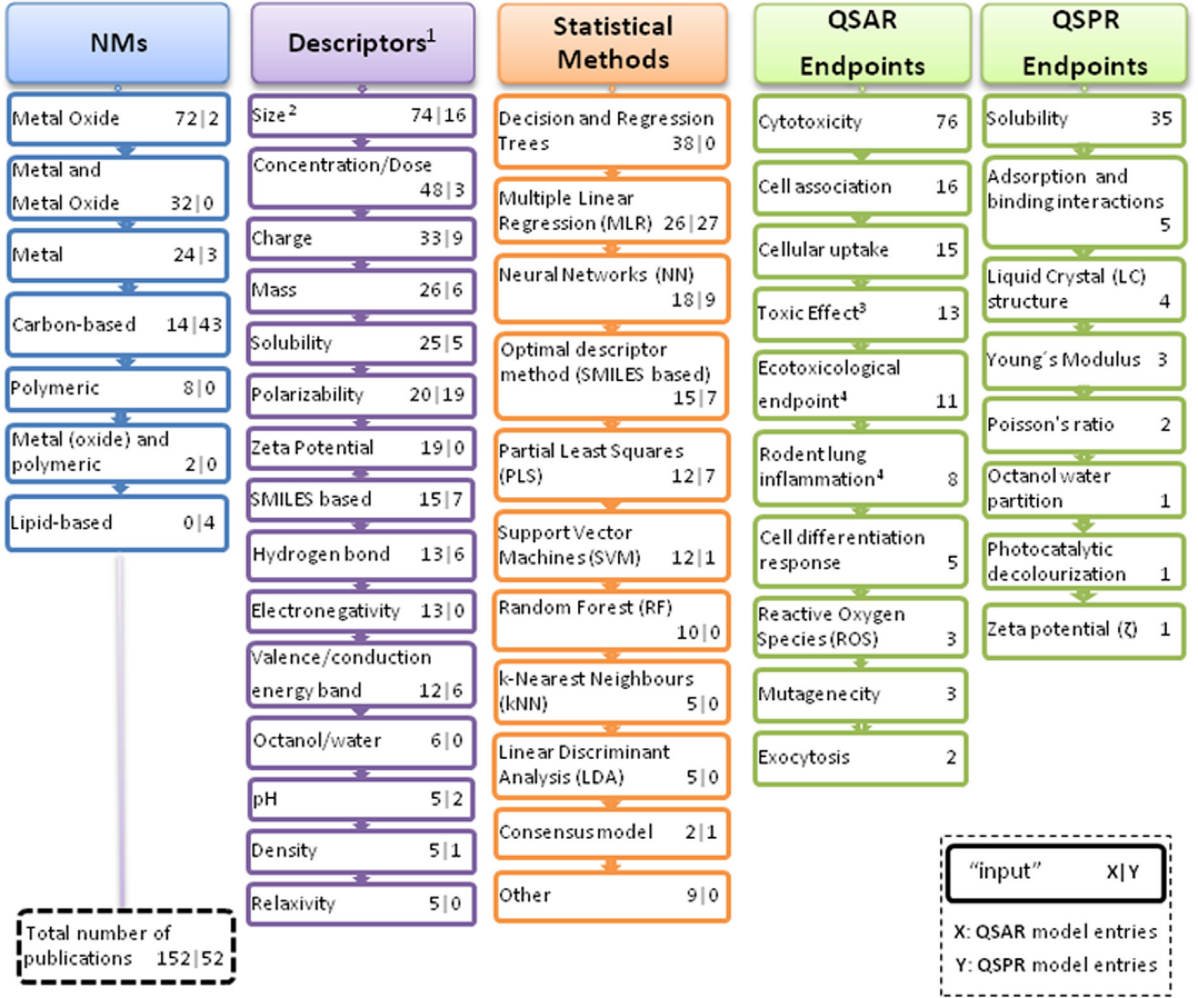
Summary of the landscape of QSAR and QSPR models. Numbers assigned to QSAR/QSPR models quantify the specific weight of each element within the same type of model. For example, the number of models applicable to carbon-based MNs corresponds to 14 and 43 for QSAR and QSPR models, respectively. The total numbers of QSAR and QSPR models in the inventory are 152 and 52, respectively). ^1^Most representative descriptors were included. ^2^Size descriptor includes size, radius, diameter, length, volume and aggregation/agglomeration. ^3^Endpoint defined by the author. ^4^Endpoint defined to group highly associated endpoints.

Models found in the literature search cover a total of 44 different MNs, including metals, metal oxides and carbon, polymeric and lipid-based particles. As shown in Fig. 3 QSAR models for metal oxides (in particular, ZnO, Fe_2_O_3_ and TiO_2_) account for 72 out of 152 models, and 32 models included both metals and metal oxides MNs. Carbon-based and polymeric MNs are applied in only 14 QSAR models. Silica-based MNs were not identified, but two QSAR-like models that are applicable to silica are referred to in the Discussion.

Regarding the descriptors used in QSAR models, MN size, either determined from electron microscopy images or determined in different relevant liquid exposure media as water, PBS or Dulbecco modified eagle medium (i.e. hydrodynamic diameter typically obtained by light diffraction scattering) appears as the most common geometric descriptor (90 out of 204) used in the reviewed models [19], [20], including primary or aggregated size (e.g. [21]) or volume (e.g. [22]).

Most QSAR models populating the inventory predict effects of MNs by means of *in vitro* cytotoxicity studies in different cell types, accounting for 152 out of 204 models, in agreement with the vast body of literature that exists examining the potential effect of MNs in *in vitro* experiments. Cytotoxicity (calculated as LC_50_, EC_50,_ which are the effective concentrations that kill or inhibit 50% of the living systems, respectively) [23] and membrane damage [24] are common predicted endpoints. Due to the large variety of *in vitro* studies, some authors (6 out of 152) have defined a composite endpoint such as “biological activity” [25], [26], or “toxic effect”, as endpoints determined by aggregating different related response measures [27].

In addition, an “ecotoxicological endpoint” is defined as an aggregation of different ecotoxicity-related endpoints such as LC_50_ [19], or percentage of mortality [28] and is found in 11 models (Fig. 3). In ecotoxicology, more *in vitro* tests are available supporting model development than *in vivo* tests (35 versus 24).

Regarding QSPR models, 43 out of 52 are built on carbon-based MNs (i.e. fullerenes and carbon nanotubes). Thirty manuscripts focus on solubility of C60 in different organic solvents [29], [30], [31], [32]. In addition, CNTs are considered in 12 manuscripts.

In contrast to carbon-based MNs, metal and metal oxide MNs only account for 5 out of 52 QSPR models. Only one study applied to inorganic MNs is available in the inventory, predicting Zeta potential from a set of 18 metal oxides MNs. This is one of the few attempts to predict a nanospecific property by the weighted energy of the highest occupied molecular orbital (quantum mechanical theoretical descriptor) and the spherical size of the MNs, which is a descriptor generated from TEM images [33]. It is also worth recalling that zeta potential has been frequently used as a descriptor in different QSAR studies (Fig. 3) [34], [35], [36]. Other endpoints predicted by QSPRs are adsorption and binding interactions [37] and the octanol-water partition coefficient [38].

#### 3.3.2 Availability of environmental exposure models for MNs

In this section, we report the mathematical models applied to calculate PECs, which can be used in chemical risk assessments. Two main different types of models are available in the literature to this aim: (1) mass flow models that typically track the materials from production and manufacturing to use and end-of-life stages, identifying at each stage how much material is released into which technical or environmental compartment, and (2) environmental multimedia fate models^3^. These study the fate and transport of MNs (transfer between compartments, advection and deposition fluxes) in a system by modelling physicochemical processes, such as agglomeration and sedimentation.

**Fig. 4:**
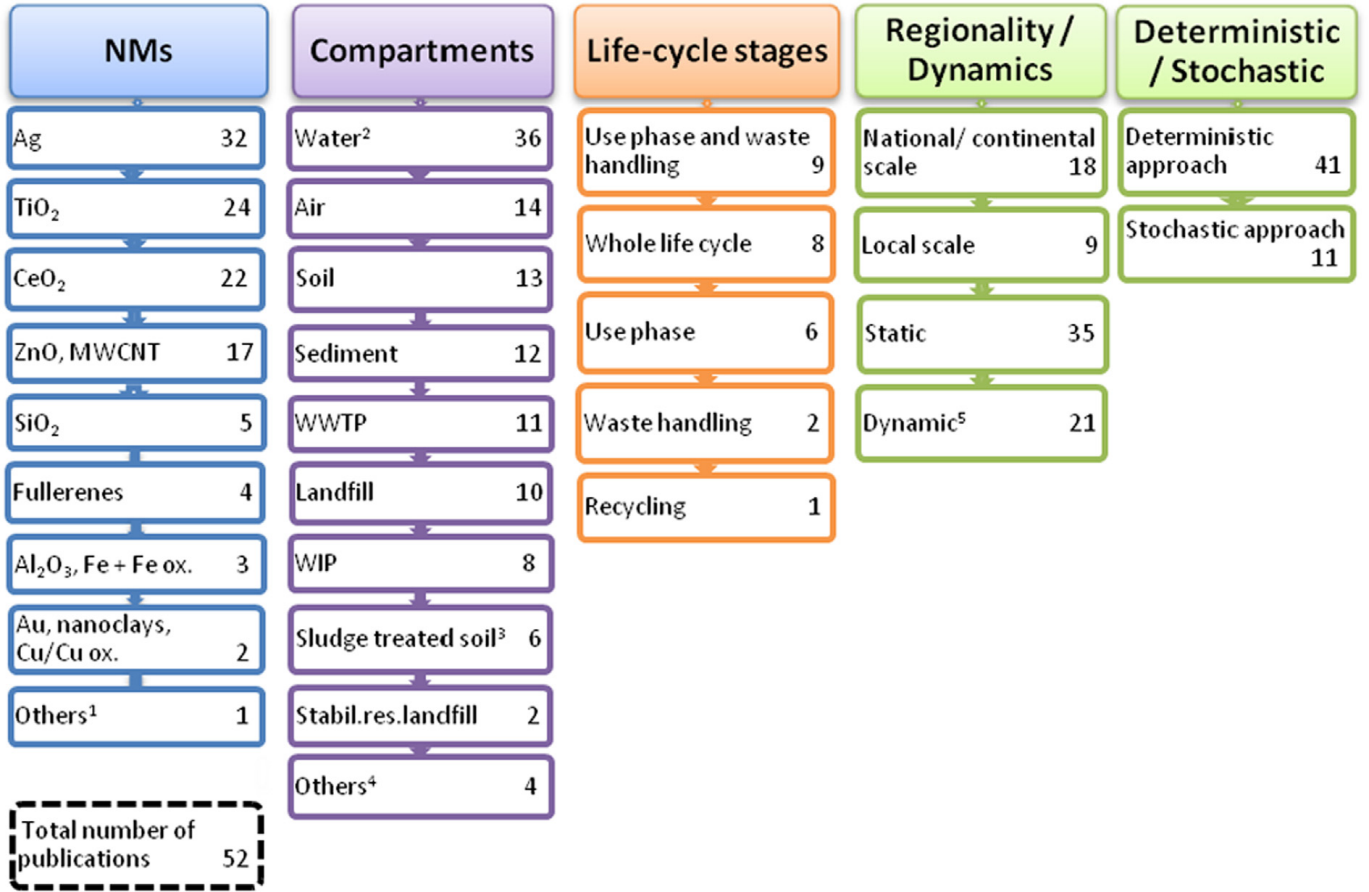
Summary of the landscape of environmental exposure models. The total number of models corresponds to the total number of entries in the inventory (52). ^1^MNs include organo-silica, hydroxyapatite, latex, CuCO_3_, quantum dots, carbon black, Ca peroxide, keratin fibers and Al. These MNs only appear once in the inventory. ^2^Includes surface water, rivers, natural freshwater and drinking water. ^3^Also including agricultural soil. ^4^Include incinerated ash landfill, groundwater, drinking water plant, human body, lungs, swimming pools and bioactive landfill, marine biota, vegetation and agricultural soil. ^5^The dynamic modelling of release describes the evolution over time of the amounts of MNs released to the environment, including also dynamic model outputs.

As shown in Fig. 4, Ag was included in 32 out of the 52 publications reported. TiO_2_ (24), CeO_2_ (10) were the most frequently evaluated metal oxides and the most studied carbon-based MN was MWCNT, which was represented in 10 studies. The total number of MNs covered in the inventory is 21.

Mass flow models reported in the inventory consider the mass of MNs, and do not consider physicochemical properties as inputs. Arvidsson et al. [39] reported a simple particle flow analysis approach using three different case studies: TiO_2_ in sunscreen and Ag in textiles and circuit electronics. To determine particle number, representative sizes obtained from literature and company websites were assigned to both MNs, and release to the environment (compartments were not specified) was estimated qualitatively based on different factors such as technology diffusion, consumption per capita of nano-products or product lifetime. The study concluded that most nano-TiO_2_ was released from sunscreen use.

Although mass flow models attempt to estimate MN concentrations in environmental compartments, such estimates are in general not based on fundamental fate and transport analysis. Because data is lacking on MN transfers to and from the compartments considered in mass flow models [40], the environmental parameters are usually defined following worst case scenarios, e.g. no sedimentation [39], or no transformation or deposition in rivers [41]. These different scenarios try to address the uncertainties associated with the behaviour of MNs in the aquatic environment. Dimensionless transfer coefficients are applied to determine MN transfer from one compartment to another. As for release factors, such coefficients are commonly obtained from literature sources when available or by expert judgment.

Environmental fate multimedia models describe the behaviour of MNs considering transformation and degradation processes (e.g. dissolution), interaction with suspended particulate matter (i.e. heteroaggregation) and transport processess (e.g. sedimentation). In this manuscript we consider multimedia model types, or models developed for the aquatic compartment, because these are more relevant for REACH applications, but the inventory is also populated with models specifically developed for the soil compartment.

MNs covered by environmental fate process-based models are limited to a restricted number of metal oxides (12, 9 and 5 out of 24 studies for CeO_2_, TiO_2_, and ZnO, respectively) and Ag, which was included in 11 out of the 24 publications included in the inventory. The PEC of other MNs (CNT, Cu, Cu oxides, Fe, Al_2_O_3_, nanoclays and SiO_2_) in different environmental compartments was evaluated in only one study [42].

Two large environmental fate models have been reported: i) SimpleBoxNano (SB4N) [43], [44] and ii) MendNano [42], [45]. Both models consider the environment as a collection of well-mixed compartments, each representing a specific medium or biological entity, with intermediate mass transport between compartments. They include aggregation/agglomeration^4^, hetero-aggregation, sedimentation, dissolution and transformation reactions in addition to transport affecting MNs bound to particulate matter. One of the main differences is that SB4N considers first order rate constants to estimate transport and transformation processes, while MendNano assumes time-independent partitioning ratios for processes of aggregation, to account for colloidal behaviour.

Thermodynamic equilibrium does not apply to MNs [46], thus SB4N proposes different forms (species) for MNs in the different compartments: i) freely dispersed, ii) hetero-agglomerated with natural colloidal particles (<450 nm), or (3) attached to larger natural particles (>450 nm), which are subjected to gravitational forces (sedimentation). Apart from this concept, other two elements are actually included in SB4N compared to the model addressing conventional chemicals: i) transformation processes are considered as altered species of the same MNs (i.e. not considered a removal process) and ii) dissolution (release of ions from the NM surface) is applied as a removal process. In air, behaviour of MNs is interpreted via the aerosol coagulation, where first-order rate constants for “aggregation” and “attachment” are applied.

Differences in the PEC estimated by mass flow models and multimedia environmental fate models depend on the environmental compartments considered. For instance, SB4N [43], [44] shows that atmospheric deposition is a relatively effective removal process, since PEC for TiO_2_ in air was 170 times smaller than PEC calculated in [47], and that steady-state is reached within one year of study. On other hand, PECs estimated in the water compartment were of the same order of magnitude, revealing that removal by sedimentation of MNs did not lead to significant differences between both models.

Other fate models in aquatic media with different degrees of complexity have been developed, as extensively reviewed by Nowack [48].

## 4 Discussion

In this manuscript we have developed model templates to systematically and consistently report different types of computational models relevant in the risk assessment of MNs. In particular, we have updated the QMRF to report QSARs and QSPRs applicable to MNs, and we have developed a reporting standard for PBK and environmental exposure models. In addition, we have populated these model templates with 152 QSARs, 52 QSPRs, and 52 environmental fate models, creating MN-specific model inventories.

The discussion on refining the QMRF to apply to MNs is ongoing, and it is reflected by the availability of QSARs and QSPRs [7]. In our reporting template, the sections specific to MNs include information on their physicochemical properties (e.g. shape, coating, size). In developing the PBK/environmental exposure model template, a challenge was to report kinetic models of different types, while also keeping the reporting template as a simple format, by selecting the most relevant information for regulatory application.

The application of the reporting standards to existing models generated a model inventory that reflects the current landscape for different MN modelling fields related to risk assessment.

In the following paragraphs, we summarise the relevance of the reported models to support different aspects of MN risk assessment, with particular reference to REACH requirements. We focus on the applicability domain of the models, on how the outcomes of the models match parameters of regulatory relevance, on the type of datasets used in model development and validation, and on the main assumptions and uncertainties.

### 4.1 Model applicability domain

The OECD adopted the concept of applicability domain for defining the limits of validity of a QSAR (e.g. types of chemical structures, physicochemical properties and mechanisms of action) [3]. Predictions for substances falling in the applicability domain are expected to be more reliable. There are several ways to determine the applicability domain of QSARs [49], each of them having advantages and disadvantages [50], [51]. Buser et al. [14] highlighted the need to report the applicability domain for exposure models. This information is included in the model reporting template.

The applicability domain for the QSAR/QSPR models reported in the inventory is not always explicitly defined in their respective publications. In the case of some models, the applicability domain is defined quite narrowly in terms of the NM type. Other models are more generally applicable to different types of NM. For example, a generic approach referred to as “perturbation modelling” has been used to predict the cytotoxicity of silica, nickel, and nickel(II) oxide nanoparticles [52], [53] on the basis of physicochemical descriptors and the experimental conditions of the study. These “QSAR-like” models were not identified in the literature review and were therefore not included in the Nanocomput QSPR/QSAR inventory.

Only a few of the environmental exposure models in the inventory reported their applicability domain. For instance, Bachler et al. [54] defined the applicability domain as “ionic silver and 15–150 nm silver nanoparticles, which were not coated with substances designed to prolong the circulatory time (e.g., polyethylene glycol)”. Li et al. [55] stated that the model can be applied to “non-degradable/non-metabolisable nanoparticles”. In most cases, however, the applicability domain was not reported.

### 4.2 Use of datasets and descriptors in the development of QSAR/QSPR models

Regarding the datasets available for model building, as few as 47 out of 204 models in the inventory used their own generated experimental data for model development [56], [57], [58]. Thus most studies in the inventory are limited to small datasets, obtained by the same research group under matching conditions. It also appears that a limited number datasets have been used extensively in a large number of publications [26], [58], [59], [60], [61].

From the descriptors selected for model building, 137, 72 and 77 out of 204 models reported particle size, coating/functionalization and shape, respectively. This revealed that physicochemical characterisation of MNs is not routinely available in (eco)toxicological studies. In other cases, descriptors were generated computationally by Molecular Dynamics simulations [62] or with software tools such as DRAGON [63].

Access to open and curated databases would support model development. This is the objective of the ongoing H2020 projects GRACIOUS (https://www.h2020gracious.eu/), NanoCommons (https://www.nanocommons.eu/) and NanoReg2 (http://www.nanoreg2.eu/about).

### 4.3 Assumptions and uncertainties

One of the most critical points in the environmental exposure models is the approach used to determine how MNs reach the different environmental compartments. The first difficulty relies on the very little information available from the manufacturer regarding the incorporation of MNs in commercial products, making it very difficult to determine the degree of market penetration. [47], [64]. One of the approaches, first proposed by Nowack and co-workers, has been widely used by different authors with slightly different methodologies: i) first, worldwide (an at a country level when available) production volume is allocated to different countries/regions by means of the population of the industrialised world [47], in proportion to Gross Domestic Product [65] or by the Inequality-adjusted Human Development Index (IHDI, which gives an idea of human development achievement) [66]; ii) second, production volume is allocated to different product categories (e.g. paints, coatings, electronics, textile) based on internet sources and also from knowledge about NM concentrations in the different nano-enabled products. It is commonly assumed that the allocation of different MNs to different products follows the same pattern for several MNs and that available models are mostly applied to MNs that are considered spherical and uncoated. It is important to take into account that all these assumptions bring uncertainty to the final assessment.

Environmental exposure model simulations reported in the inventory are not generally based on measurements available to compute or validate model outputs, e.g. the agglomeration/deposition of MNs or monitored environmental concentrations. To compensate for the lack of data on real MN concentration in the environment, research on detection of MNs from background material is ongoing [67]. In general, it is observed that environmental exposure models consider that all compartments are well mixed (homogeneous) and that the rates of mechanical transport are independent of chemical composition and crystal form [68].

### 4.4 Relevance for REACH

Only 6 QSPRs predict MN properties that are required for REACH, i.e. 1 for water solubility [38], 1 for octanol-water partition coefficient [38], 4 dispersion in organic solvents [69], [70], [71], and 1 adsorption/sorption [37].

The inventory reports 5 models for ecotoxicological endpoints. Of these, only 1 model reports algorithms predicting REACH-relevant endpoints, as the others suggest new biological metrics that integrate multiple toxicological endpoints [27], [28], [72], [73], [74]. Chen et al. [19] reported a series of global and species-specific models that could be applied to predict REACH endpoints. QSAR models build on EC50 and LC50 values for *Danio rerio*, *Pseudokirchneriella subcapitata*, *Daphnia magna* and *Staphylococcus aureus*.

There are no models covering the REACH toxicological endpoints on acute toxicity, repeated dose toxicity, (skin and respiratory) sensitisation, carcinogenicity, and reproductive toxicity. Most of the QSARs predicting human hazard were actually developed for cytotoxicity endpoints, which are not of direct relevance. In the inventory, a QSAR predicting mutagenicity of carbon-based MNs is reported, but it is based on the Ames test, which is not considered applicable to MNs [75].

SB4N [43] is an adaptation of the SimpleBox model, and provides as an output PECs at the steady state. SimpleBox has been used as a regional distribution module in the EU system for Evaluation of Substances (EUSES) model, which is applied in environmental exposure assessment in REACH [9]. However, the lack of analytical techniques able to measure trace concentration of MNs and differentiate between background and MNs, hinders the validation of such models. The SB4N model consists of around 70 inputs and some of them are nanospecific, such as size, density, attachment efficiency or dissolution rate. It is recognised that more experimental data are needed for some of the parameters that are not fully covered by existing colloidal theory (e.g. hetero-attachment efficiency) [76]. Furthermore, for a better description of aggregation phenomena, fractal modelling can complement existing kinetic fate models by providing more accurate integration of shape, structure, density and collision efficiency [77].

## 5 Conclusions

Taking into account international discussions on the need for transparent and systematic model reporting, we have presented two different templates that allow the reporting of QSARs and QSPRs, as well as PBK and environmental exposure models, which are relevant in different phases of risk assessment. The templates are tailored to the reporting of MNs, but can also be applied to all types of chemicals. Whereas the QSAR/QSPR reporting template is based on the QMRF previously developed for chemicals, the PBK/environmental exposure reporting template was developed from scratch.

We have complied and described model inventories derived from a comprehensive literature search of MN-relevant predictive models and by using the templates to capture key model characteristics. Analysis of these inventories reveals how the models may prove useful in regulatory risk assessments, by classifying the models in terms of their outputs and by declared uncertainties and assumptions. Using key descriptors from the reporting templates, we have also shown how these inventories can give an overview of the landscape of available models for MNs. We found that only one QSAR is predicting REACH-relevant ecotoxicity endpoint [25], whereas toxicological endpoints on acute toxicity, repeated dose toxicity, (skin and respiratory) sensitisation, carcinogenicity, and reproductive toxicity are not covered in the QSAR inventory.

The environmental exposure models landscape identified tools that can support risk assessment [41], [42], [43], [44]. However, the lack of analytical techniques supporting the validation of these models hinders their validation, application and acceptance in risk assessment.

## Footnotes

1 In this manuscript we refer to the whole process of chemicals risk assessment to include both domains of mathematical models covering hazard identification and assessment (QSARs) and exposure assessment (PBK, environmental fate models)

2 Files are downloadable as *S1: Inventory of Quantitative Structure-Property Relationship (QSPR) and Quantitative Structure-Activity Relationship (QSAR) models (Excel workbook)*, *S2: Model inventories for physiologically based kinetic (PBK) models, dosimetry models and environmental fate models (Excel workbooks)*. QSAR/QSPR model inventory is also available as a document format in .pdf *as S3: Individual QSPR/QSAR model descriptions in document format (pdf file)*.

3 In the inventory, process-based environmental fate models were evaluated in detail, including models assessing all compartments as well as models specific for fate in the aquatic and soil compartment. Models developed specifically for the soil compartment are not analysed in this manuscript. More information is available in Worth et al. [17].

4 Agglomeration is defined as collection of weakly or medium strongly bound particles whereas aggregation refers to a particle comprising strongly bonded or fused particles held together by strong forces such as covalent bonds. In fact, modelling language does not distinguish between the two because often it is assumed that once an agglomerate is formed, it does not de-agglomerate [76]. As a result, the two terms are used interchangeably.
